# An optimized sandwich ELISA for quantitative detection of fowl adenovirus serotype 4 (FAdV-4) in chickens

**DOI:** 10.1016/j.psj.2025.106259

**Published:** 2025-12-12

**Authors:** Huimin Ma, Yuhang Zhou, Shipeng Wang, Xiangyu Xie, Ruiji Chen, Qi Zheng, Lisha Zha, Xinyue Chang

**Affiliations:** a College of Veterinary Medicine, Anhui Agricultural University, Hefei 230036, PR China; b School of Biomedical Sciences, Hunan University, Changsha 410082, PR China

**Keywords:** FAdV-4, Sandwich ELISA, Fiber 1 protein, Monoclonal antibody (mAb)

## Abstract

Fowl adenovirus serotype 4 (FAdV-4) causes hydropericardium-hepatitis syndrome (HHS) in chickens, resulting in severe economic losses worldwide. Fast and accurate diagnostic methods for FAdV-4 are required to monitor its prevalence and provide plausible strategies for prevention. Sandwich ELISA is a common and rapid diagnostic technique; however, an FAdV-4 ELISA kit is not commercially available in China. Fiber 1 protein of FAdV-4 has been used as diagnostic targets. In this study, we firstly screened 5 monoclonal antibodies (mAbs) against fiber 1 from rabbits using phage display. ELISA and indirect fluorescence assay (IFA) showed these mAbs specifically bound to both fiber 1 and FAdV-4. Binding affinities of the mAbs forming optimal pair were lower than 10^−12^ M, which recognized the knob and tail domain of fiber 1 by docking simulation, respectively. Next, sandwich ELISA for FAdV-4 was optimized, demonstrating excellent specificity, repeatability and reproducibility. The assay could detect as low as 0.532 ng/mL fiber 1 protein. For FAdV-4 diagnosis in clinical sera, the results of ELISA showed 97.44 % consistency with qPCR. Additionally, viral antigen levels in chick tissues determined by ELISA mirrored the viral loads by qPCR. The FAdV-4 kinetics in chicks quantified by ELISA and qPCR showed high consistency. In conclusion, we successfully developed a sensitive and specific ELISA for FAdV-4 diagnosis and quantification, offering a potential diagnostic tool in veterinary clinic.

## Introduction

Inclusion body hepatitis (IBH) caused by fowl adenovirus (FAdV) was firstly detected in Taiwan province of China as early as in 1976, which thereafter spread throughout the mainland of China (Niu et al., 2022). Since 2015, FAdV outbreaks consecutively occur in China, leading to substantial economic losses in poultry industry (Li et al., 2016). FAdV has been categorized into 12 serotypes, namely 1, 2, 3, 4, 5, 6, 7, 8a, 8b, 9, 10 and 11, amongst which the highly pathogenic FAdV-4 is dominantly circulating in China in recent years (Li et al., 2016; Ye et al., 2016; Niu et al., 2022). FAdV-4 mainly causes hepatitis-hydropericardium syndrome (HHS) and hydropericardium syndrome (HPS) in chickens, manifesting clear and straw-colored fluid accumulation in pericardial sac, liver necrosis and lethargy (Rashid et al., 2024). Studies have demonstrated that FAdV-4 affects both broiler and layer chickens, mounting up to 100 % mortality rates in chickens (Dai et al., 2024; Pouladi et al., 2024). In addition to chickens, other fowls including ducks (Tang et al., 2022), geese (Wu et al., 2024), and cranes (Xue et al., 2023) could be infected by FAdV-4 as well, aggravating the prevalence and hindering the prevention of the virus. Therefore, seeking a rapid and accurate diagnostic method for FAdV-4 is the premise to mitigate the influence of the virus.

Currently, quantitative real-time PCR (RT-qPCR) is the standard technique to detect FAdV-4. During the past years, more PCR-based approaches targeting genes of FAdV-4 have been developed. For example, three different rapid and effective multienzyme isothermal rapid amplification (MIRA) assays targeting at hexon gene were reported for field detection of FAdV-4 (Dai et al., 2024). In addition, a CRISPR/Cas12a system based on loop-mediated isothermal amplification (LAMP) was developed, which showed high consistency with RT-qPCR (Yu et al., 2024). Both methods rely on isothermal amplification, which is convenient and rapid in the field. However, PCR-based methods might overestimate the viruses because their targets are viral DNA, which might exist in chickens after recovery (Schachner et al., 2014; Li et al., 2016). In this case, methods aiming at detecting viral proteins are excellent options, such as sandwich ELISA. Monoclonal antibody-based sandwich ELISA methods for FAdV-4 detection demonstrated high efficiencies (Shao et al., 2019a, 2019b). Nevertheless, commercial ELISA kits for FAdV-4 are still not available.

As a member of *Adenoviridae* family, FAdV-4 viral particles, composing of penton and hexon structural proteins, exhibit icosahedron symmetry with antenna-like fibers protruding out of the particles. The hexon is highly conserved among avian adenoviruses, which is often used as gene detection target (Niu et al., 2018; Kang et al., 2025). Different from most FAdVs, FAdV-4 virions contain two different fiber proteins, i.e. fiber 1 and fiber 2, both of which contribute to the viral pathogenicity. Fiber 2 protein plays a critical role in virulence of FAdV-4 strains in China (Zhang et al., 2018), therefore attenuated live vaccine candidates were developed with edited or deleted fiber 2 protein (Xie et al., 2021, 2022). The fact that fiber 2-deleted FAdV-4 was a potential vaccine candidate implies the fiber 2 was dispensable for FAdV-4 survival. On the other hand, fiber 1 gene was shown to be essential for FAdV-4 survival in cells (Zou et al., 2021). More importantly, fiber 1 protein directly binds to the coxsackievirus and adenovirus receptor (CAR) homology, indicating the interaction between fiber 1 and CAR initiates the viral invasion (Pan et al., 2020). It has been shown that antibodies against the fiber 1 knob domain efficiently block FAdV-4 infection. Therefore, fiber 1 is vital for FAdV-4 infection and survival, which is suitable to be the protein target for FAdV-4 diagnosis. Furthermore, fiber 1 protein, especially the knob domain, showed relatively higher antigenicity (Wang et al., 2020). These advantages of fiber 1 make it an excellent protein target for ELISA development to detect FAdV-4.

Although commercial vaccines are available in China, FAdV-4 is not cleared and still gives rise to epidemics periodically (Schachner et al., 2018; Wang et al., 2023). Highly sensitive and efficient diagnostic tools for FAdV-4 are required. Sandwich ELISA, based on a pair of monoclonal antibodies (mAbs), has been widely used in clinic and farming diagnosis due to the advantages of accuracy, sensitivity and easy-handling (Civera et al., 2022). Compared to the classic hybridoma technique, phage-display technique directly disclosed the variable region sequences of mAbs, greatly simplifying the mAbs screening process. Additionally, several rounds of panning in phage-display technique could vastly increase the probability to obtain high-affinity mAbs (Hammers and Stanley, 2014; Mahdavi et al., 2022).

In this study, we firstly screened rabbit-derived mAbs against fiber 1 protein using phage-display and set up a pair of mAbs for sandwich ELISA. Then, a sandwich ELISA method was established and optimized, which exhibited excellent repeatability and reproducibility. The established assay successfully identified FAdV-4 in clinical serum samples. Furthermore, the FAdV-4 infection kinetics in chicks were measured, which was highly consistent with the results of qPCR. The present work provides an exceptional alternative for FAdV-4 diagnosis in clinic and the field.

## Materials and methods

### Viruses, cells and vectors

FAdV-4 strain (AH-FAdV-4) and helper phage M13K07 were stored in our laboratory. Other strains of FAdV, such as FAdV-1, FAdV-8a and FAdV-8b were kindly provided by Prof. Yuchen Nan from Northwest Agriculture & Forestry University. DAdV-3 was kindly provided by Prof. Fangfang Chen from Anhui Agricultrural University. CAV was kindly provided by Prof. Xuelan Liu from Anhui Agricultrural University. ALV was purchased from China Institute of Veterinary Drug Control. LMH cell line for FAdV-4 replication, HEK293F cells for antibody production and Sf9 cells for fiber 1 protein production were kept in our laboratory. Phage vector pComb3XSS and eukaryotic expression vector pTT5 were kept in our laboratory.

### Production of fiber 1 protein

Fiber 1 protein was expressed in baculoviruses generated using Bac-to-Bac Baculovirus Expression System (Invitrogen, Shanghai, China) according to the manual. Briefly, the fiber 1-encoding gene linked with 6 × His-tag was assembled into donor plasmid pFastBac 1, which was then transformed in DH10Bac cells for Bacmid recombination. After two rounds of blue colony picking, the recombinant bacmid containing fiber 1 gene was extracted and then Sf9 cells were transfected with the bacmid. Next, baculoviruses were enriched by passaging in fresh Sf9 cells. Finally, the cell supernatants containing fiber 1 protein were collected. The fiber 1 protein was then purified using Ni^2+^ resin (Genscript, Nanjing, China), which was characterized by SDS-PAGE and Western blot. The concentration of purified fiber 1 protein was determined using BCA (Beyotime Biotechnology, Shanghai, China).

### Immunization of rabbits

New Zealand White rabbits (6-month-old) were purchased from Experimental Animal Center of Anhui Medical University and kept in separate cages at ambient temperature with sufficient clean food and water. Animals were adjusted themselves to the facility for a week before experimentation. All animal experiments were approved by the Animal Care and Use Committee, Anhui Agricultural University (AHAU 2022043). All animal handling and experiments were performed strictly in accordance to the guidance and regulations of the Animal Care and Use Committee, Anhui Agricultural University. Four rabbits were immunized with 1 × 10^5^ TCID_50_ heat-inactivated FAdV-4 formulated in 1 mL complete Freund’s adjuvant (Makewonderbio, Beijing, China) via subcutaneous injection at day 0. On day 7, 14, and 21, rabbits were subcutaneously injected with 5 × 10^4^ TCID_50_ heat-inactivated FAdV-4 formulated in 0.5 mL incomplete Freund’s adjuvant (Makewonderbio, Beijing, China). Serum samples were collected at day 7, 14, 21, and 28 from ear vein. Rabbits were euthanized by CO_2_ inhalation on day 30, after which the spleens were harvested.

### ELISA

To examine the antibody titers of immunized sera, ELISA was conducted as previously described (Zha et al., 2021). Basically, 1 μg/mL fiber 1 protein in PBS was coated on plates at 4°C overnight. Then, PBS-1% Casein was added to block the plate for 2 hours at room temperature. Subsequently, serum samples were added and serially diluted in PBS-1% Casein for 1 hour at room temperature. Lastly, detection antibody goat anti-rabbit IgG-HRP (Beyotime Biotechnology, Shanghai, China) was added and incubated for 1 hour at room temperature, after which the TMB development solution and stop solution were added sequentially. The OD_450 nm_ was read immediately and titers were determined as the dilution fold that the OD_450 nm_ value was higher than 2.1-fold of the negative control (day 0 serum) (Zhao et al., 1991; Liu et al., 2010).

### V_H_/V_L_ gene and single chain variable fragment (ScFv) amplification

The spleens were homogenized and erythrocytes were removed by incubating the splenocytes in ACK buffer at room temperature for 5 min, which were then resuspended in PBS. Total RNA of 1 × 10^7^ splenocytes was extracted using Universal RNA Mini Kit (HANGZHOU BEIWO Medical Technology, Hangzhou, China), which was immediately reverse transcribed into cDNA using HyperScript III 1^st^ Strand cDNA Synthesis Kit (EnzyArtisan, Shanghai, China). Then, V_H_ and V_L_ genes were amplified by PCR using primer sets listed in Table 1 as follows: 25 μL KOD One PCR Master Mix, 1 μL V_H_-FP/ V_L_-FP, 1 μL V_H_-RP/ V_L_-RP, 3 μL cDNA, 20 μL ddH_2_O. The PCR products were loaded in agarose gel and bands around 400 bp were cut and purified. Overlap PCR was performed using purified V_H_/V_L_ products and primers (F: GAGGAGGAAAAAGAGGCCCAGGCGGCC; R: GAGAGGAGGCTATTGGCCGGCCTGGCC) to amplify scFv.

**Table 1:** Primer sequences for V_H_ and V_L_ amplification.

Primer	Nucleotide sequence (5′−3′)
V_H_-FP	GGTGGGGGTGGTTCCTCTAGATCTTCCCAGTCGKTGGAGGAGTCC
	GGTGGGGGTGGTTCCTCTAGATCTTCCCAGWCAGTGAAGGAGTCC
	GGTGGGGGTGGTTCCTCTAGATCTTCCCAGTCGCTGGRGGAGTCC
	GGTGGGGGTGGTTCCTCTAGATCTTCCCAGTCGGTGGAGGAGTCC
V_H_-RP	AGGAGGCTATTGGCCGGCCTGGCCGCAGCAGGGGGCCAGTGGGAAGACTGACGGAGC
	AGGAGGCTATTGGCCGGCCTGGCCTGARGAGAYGGTGACCAGGGTGCC
V_L_-FP	AAAAGAGGCCCAGGCGGCCGAGCTCGTGMTGACCCAGACTCCA
	AAAAGAGGCCCAGGCGGCCGAGCTCGATMTGACCCAGACTCCA
	AAAAGAGGCCCAGGCGGCCGCYCAAGTGCTGACCCAG
	AAAAGAGGCCCAGGCGGCCGCCCWAGTGATGACCCAG
V_L_-RP	GGAAGATCTAGAGGAACCACCCCCACCACCGCCCGA GCCACCGCACCTTTGATTTCCACATTGGTGCC
	GGAAGATCTAGAGGAACCACCCCCACCACCGCCCGA GCCACCGCACCTAGGATCTCCAGCTCGGTCCC

### ScFv phage library construction and panning

The scFv was ligated to *Sfi* I-digested pComb3XSS vector using T4 ligase. Next, the formed pComb3XSS-scFv was transformed into *E. coli* ER2738 competent cells, which were plated on Ampicillin^+^ plates. All colonies were harvested in fresh medium and incubated with helper phage M13K07 at room temperature for 30 min. Then, the phage-bacteria mixture was cultured at 30°C overnight in Kanamycin^+^ medium. Subsequently, 42 % (v/v) PEG/NaCl solution was added to the culture. After mixing thoroughly, the mixture was placed still on ice for 1 hour to precipitate phages. The precipitated phages were collected by centrifuging the mixture at 10000 r/min, 4°C for 20 min. The phage-containing pellets were resuspended in PBS and the precipitation procedure was repeated. Finally, the resuspended phages were stored at 4°C.

To screen the phages that display fiber 1-specific scFv, 96-well plates were coated with 1 μg/mL fiber 1 protein overnight at 4°C. After blocking plates with PBS-1% Casein at room temperature for 2 hours, the phage suspension was added to plates and incubated at room temperature for 1 hour. Then, 100 mM Glycine-HCl buffer (pH 2.0) was added and incubated at room temperature for 15 min to elute the bound phages. To preserve the activity of phages, Tris-HCl (pH 9.0) was used to neutralize the Glycine-HCl buffer. Then, ER2738 competent cells were transformed with collected phages, after which single colonies were picked and cultured at 37°C, 180 rpm for 5 hours. The phage-containing culture was then incubated on ELISA plates coated with fiber 1 protein at room temperature for 1 hour. Subsequently, mouse anti-M13 mAb conjugated with HRP (SinoBiological, Beijing, China) was added and incubated at room temperature for 1 hour, after which TMB substrate solution was added. Lastly, stop solution (1 M H_2_SO_4_) was added and OD_450 nm_ values were read. The supernatants that ER2738 cells infected with helper phage were used as negative control. The 5 colonies that showed highest OD_450 nm_ values were picked as candidate phages.

### Expression and purification of mAbs

Plasmids in the picked colonies were extracted and V_H_ and V_L_ genes were amplified, which then were assembled into C_H_- and C_L_-containing pTT5 vectors, respectively. HEK293F cells (1 × 10^6^ cells/mL) were transfected with pTT5-V_H__—_-C_H_ and pTT5-V_L_-C_L_ and cell culture supernatants containing total IgG antibodies were collected 7 days after transfection. After filtering through 0.45 μm membrane, the supernatants were loaded to HiTrap Protein A HP column (Cytiva, Shanghai, China) and the IgG antibodies were eluted with Glycine-HCl buffer (pH 2.0). The antibodies were concentrated and buffer exchanged in PBS with 30 kD cut-off Ultra Centrifugal filter (Millipore, Shanghai, China). The purified antibodies were assessed by SDS-PAGE and the concentration was determined via BCA.

### Characterization of mAbs

The recognition and binding of mAbs to FAdV-4 were examined by ELISA and IFA. One hundred TCID_50_ AH-FAdV-4 in 100 μL PBS were coated on 96-well plates at 4°C overnight, and then plates were washed with PBS for 4 times. Plates were blocked with PBS-1% Casein at room temperature for 2 hours, after which 1 μg/mL antibodies were added and incubated at room temperature for 1 hour. Rabbit anti-His tag mAbs were used as negative control. Then, goat anti-rabbit IgG-HRP antibody was added and incubated at room temperature for 1 hour after washing with PBS-0.5 % Tween for 4 times. Finally, 100 μL TMB solution was added and same volume of stop solution was added to stop the reaction. Antibodies that OD_450 nm_ values of S/N > 2.1 were regarded as positively recognize and bind to virus.

As for IFA, LMH cells were cultured in 24-well plates until 80∼90 % confluence, which were then incubated with 200 TCID_50_ AH-FAdV-4 in 500 μL culture medium for 2 hours at 37°C. The virus was removed and 500 μL fresh medium was added to culture cells for another 48 hours. After washing cells with PBS for 3 times, 500 μL 4 % paraformaldehyde solution and 0.5 % Triton X-100 was sequentially added to fix and permeabilize cells at room temperature for 30 min, respectively. After washing with PBS for 3 times, cells were blocked with 500 μL PBS-1% Casein at 37°C for 1 hour. Subsequently, 1 μg/mL antibodies in 500 μL PBS-1% Casein were added and incubated at 37°C for 1 hour, after which goat anti-rabbit IgG-FITC antibody was added and incubated at 37°C for 1 hour. Finally, DAPI was added and washed away after incubating at room temperature in dark for 20 min. The binding of antibodies to viruses were visualized under microscopy.

### Analysis of binding between mAbs and fiber 1 protein using biolayer interferometry (BLI) and protein-protein docking

The affinities between mAbs and fiber 1 protein were determined using BLI. Firstly, the mAb was immobilized on Protein A biosensors. Then, the fiber1 protein at a serial of concentrations (10, 5, 2.5, 1.25, and 0.625 nM) was applied to the sensors, which were monitored in real-time as the association phase. Next, the sensors were immersed into 0.02 % PBST to monitor the dissociation of the bound fiber 1 protein. Finally, the association rate constant K_on_ and dissociation rate constant K_off_ were generated by fitting the association and dissociation curves. The equilibrium dissociation constant K_D_ was calculated according to K_D_ = K_off_/K_on_.

Subsequently, the 3D structures of fiber 1 protein and the variable fragments (Fv) of mAbs were modelled in AlphaFold2-multimer, which were then imported in ClusPro for protein-protein docking (Kozakov et al., 2017; Desta et al., 2020; Jones et al., 2022). The readout of docking was visualized by PyMOL (Version 2.6.0a0 Open-Source).

### Establishment of an optimized sandwich ELISA for detecting fiber 1 protein

To establish an outstanding sandwich ELISA for fiber 1 protein detection, ELISA conditions were optimized: detection antibody determination, antibody pairing, antibody working concentrations assessment, coating, blocking, sample and detection antibody application conditions. All assays were conducted according to the standard protocol as follows until the condition was optimized: 1 μg/mL fiber 1 protein or antibody was coated at 4°C overnight, after which plates were blocked with PBS-1% Casein at room temperature for 2 hours. Then, either fiber 1 protein or detection antibody was added and incubated at room temperature for 1 hour. Finally, develop and stop solution were added in order. To simplify the description, only different procedures are mentioned below. All experiments were performed in 2 independent biological replicates.

### Determination of detection antibody

The screened antibodies were firstly labeled with HRP using HRP Labeling Kit (Makewonderbio, Beijing, China) following the manufacture’s manual. After the plate was coated with 1 μg/mL fiber 1 protein and then blocked, the HRP-labeled (detection) antibodies were serially diluted and incubated at room temperature for 1 hour. The rest of ELISA was performed as the standard protocol. The antibody that showed the highest dilution times reaching half of the maximum OD_450_ values was chosen for detection antibody in following assays.

### Antibody pairing

Plates were firstly coated with 2 μg/mL capture antibody and then blocked, after which fiber 1 protein was added and serially diluted. A different HRP-labeled detection antibody was added and incubated for 1 hour. The antibody pair that showed the lowest fiber 1 concentration reaching half of the maximum OD_450_ values (OD_50_) was determined.

### Antibody working concentrations assessment

Plates were coated with different concentration of the optimal capture antibody. After blocking, 0.05 μg/mL fiber 1 protein was added and incubated for 1 hour at room temperature. Then, different concentration of detection antibody was added. PBS-1% Casein, instead of fiber 1 protein was added as negative control. The concentrations that reached maximum P/N value were defined as optimal.

### Coating condition assessment

The plates were coated with capture antibody at optimal concentration under different conditions: 4°C for 4 hours, 4°C overnight, 37°C for 2 hours, 37°C for 4 hours and 37°C overnight. After blocking, 0.05 μg/mL fiber 1 protein was added and incubated. Then, detection antibody at optimal concentration was added and incubated. PBS-1% Casein, instead of fiber 1 protein was added as negative control. The best coating condition that reached maximum P/N value was defined as optimal.

### Blocking condition assessment

After the plates were coated under optimized condition, PBS-1% Casein was added and incubated for 30, 60, 90, 120 or 150 min. Then 0.05 μg/mL fiber 1 protein and detection antibody were added sequentially. PBS-1% Casein, instead of fiber 1 protein was added as negative control. The best blocking condition that reached maximum P/N value was defined as optimal.

### Sample application condition assessment

After the plates were coated and blocked under optimal condition, 0.05 μg/mL fiber 1 protein was added and incubated for 30, 60, 90, 120, 150, 180 or 210 min. PBS-1% Casein, instead of fiber 1 protein was added as negative control. The blocking condition that reached maximum P/N value was defined as optimal.

### Detection antibody application condition assessment

After the plates were coated and blocked under optimal condition, 0.05 μg/mL fiber 1 protein was added and incubated for optimal duration. Then, detection antibody at optimal concentration was added and incubated at room temperature for 30, 60, 90, 120 or 150 min. PBS-1% Casein, instead of fiber 1 protein was added as negative control. The condition that reached maximum P/N value was defined as optimal.

### Specificity evaluation

To evaluate the specificity of the established ELISA, FAdV-4, FAdV-1, FAdV-8a, FAdV-8b, DAdV-3, CAV and ALV were applied on plates. LMH cell lysate was used as negative control.

### Sensitivity evaluation

To evaluate the sensitivity of the established ELISA, fiber 1 protein was serially diluted at 1:2 from 10 ng/mL. PBS-1% Casein was added as negative control. The sensitivity was determined as the fiber 1 concentration that P/N > 2.1.

### Repeatability and reproducibility evaluation

The repeatability was tested by applying 5 positive liver samples on the same plate. Each sample was performed for 8 replicates. The reproducibility was tested by applying 5 positive liver samples on 3 plates from different batches. The mean (X) and standard deviation (SD) was calculated and the intra-assay coefficient of variation (CV) was obtained according to CV = SD/*X* × 100 %.

### FAdV-4 diagnosis in clinical serum samples

The clinical serum samples were directly applied in the optimized sandwich ELISA to diagnose FAdV-4. The fiber 1 protein (30 ng/mL) and PBS-1% Casein were used as positive and negative control, respectively. The samples that OD_450 nm_ values of P/N > 2.1 were regarded as FAdV-4 positive. To compare the results with qPCR, genomic DNA of serum samples was extracted using DNA Viral Genome Extraction Kit (Qingdao Lijian Bio, Qingdao, China), which were used as templates in Fowl Adenovirus Type C Serotype 4 qPCR Detection Kit (XYbio, Shanghai, China).

### Quantification of viral antigen in chick tissues after FAdV-4 infection

To quantify the viral antigen, a standard curve was firstly generated. Instead of fiber 1 protein, FAdV-4 was applied on plate from 5 × 10^5^ TCID_50_/mL and serially diluted 1:3. Each titration was performed in duplicates. The antigen amounts in tissue homogenous supernatants were calculated by fitting the OD_450 nm_ value in the linear range of the standard curve. The viral antigen levels *in vivo* determined by established ELISA were equivalent to those found in FAdV-4 stocks at certain TCID_50_/mL.

Fertilized SPF eggs were purchased from Zhejiang Lihua Agricultural Co., Ltd. (Zhejiang, China) and hatched at 37.8°C with 60∼75 % humidity in an incubator for 21 days. The hatched chicks were immediately transferred to a clean and warm house to dry and fluff up, after which food and water were supplied. As soon as the chicks were fully hatched, they were maintained and handled following the guidance and regulations of the Animal Care and Use Committee, Anhui Agricultural University. The chick experiments were approved by the Animal Care and Use Committee, Anhui Agricultural University (AHAU 2024047).

To track FAdV-4 dynamics in chicks, eighteen 6-day-old SPF chicks were subcutaneously injected with 5 × 10^4^ TCID_50_ FAdV-4 (in 50 μL PBS), after which serum, heart, liver, kidney, and glandular stomach were collected from 3 euthanized chicks at 0, 4, 8, 12, 24, and 48 hours post-infection (hpi). Subsequently, 0.1 g tissue was homogenized in 500 μL cell lysis buffer (Beyotime Biotechnology, Shanghai, China) using a tissue homogenizer. The homogenized tissue was placed on ice for 30 min and then centrifuged at 10000 g for 5 min to collect the supernatants.

To examine the antigen amounts in tissues of dead chicks, five 6-day-old SPF chickens were subcutaneously injected with 5 × 10^4^ TCID_50_ FAdV-4 (in 50 μL PBS). All chicks died from infection on the third day after challenge, when tissues were collected to test antigen amounts. Genomic DNA was extracted from tissues using TRIzol reagent (Sangon Biotech, Shanghai, China) and qPCR was performed.

## Results

### Screening, expression and characterization of fiber 1-specific mAbs

**Fig. 1:**
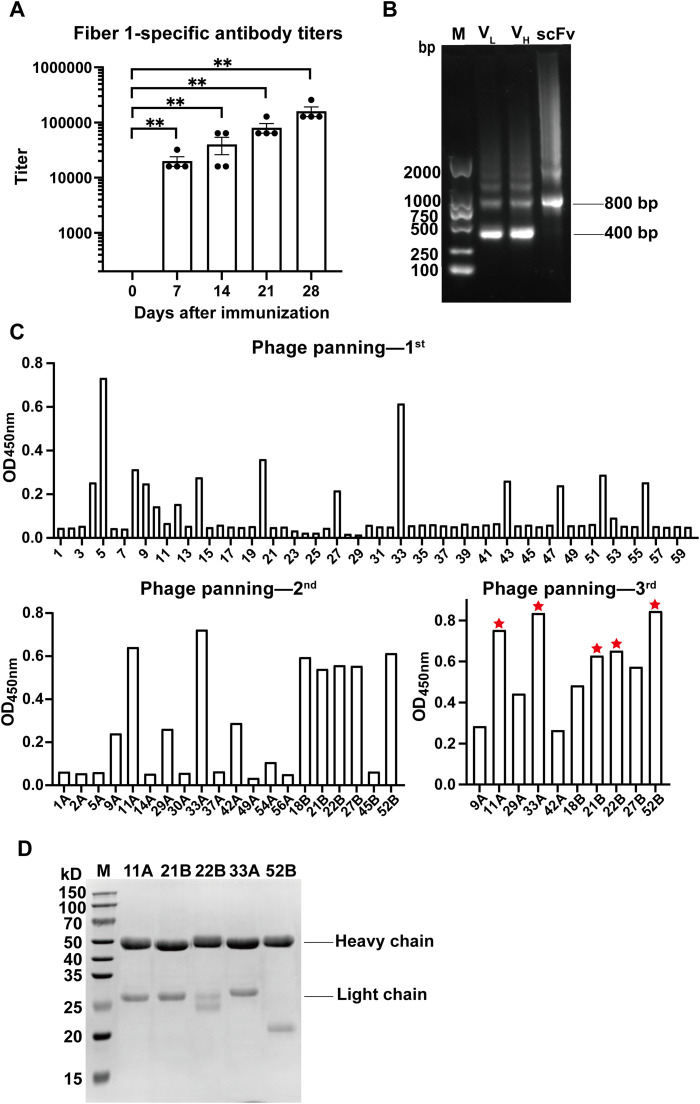
Screening and expression of fiber 1-specific mAbs. A. The fiber 1-specific antibody levels in rabbit sera on day 0, 7, 14, 21, and 28 after immunization. Each point represented one rabbit, and the error bar: ± SEM. **B.** Agarose gel images of amplified V_H_, V_L_, and scFv genes. **C.** ELISA results of three-rounds phage panning to screen fiber 1-specific mAb clones. The screened mAbs were noted by red asterisks. **D.** SDS-PAGE gel image of the purified mAbs (11A, 21B, 22B, 33A and 52B). *P* ≤ 0.01 **.

After 4 doses of immunization, the fiber 1-specific antibody levels in rabbit sera remarkably elevated. The antibody titers were as high as 1.6 × 10^5^ till day 28, which were sufficient to screen B cells from immunized spleen (Fig. 1A). To generate the scFv library, V_H_ and V_L_ genes were firstly amplified from the cDNA of splenocytes and then combined via a linker. As expected, both V_H_ and V_L_ fragments exhibited a band around 400 bp and the scFv band was 800 bp (Fig. 1B). After three rounds of phage panning, five clones 11A, 21B, 22B, 33A and 52B were identified to specifically bind to fiber 1 with relatively high affinity (Fig. 1C). Full length of the five mAbs were expressed and purified, which were characterized in SDS-PAGE gel. All mAbs displayed a heavy chain (50 kD) and a light chain (25 kD) in the gel, suggesting the mAbs were successfully expressed and purified (Fig. 1D).

**Fig. 2:**
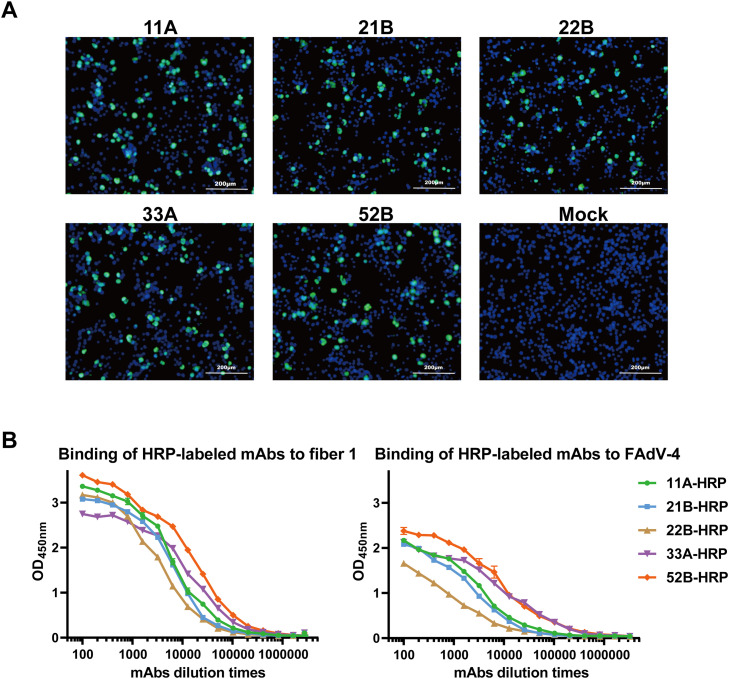
The specific binding of mAbs to FAdV-4 and fiber 1 by IFA and ELISA. A. IFA images of the five mAbs binding to FAdV-4 *in vitro*. PBS-1% Casein, instead of mAbs was added as mock control. Blue fluorescent signals illustrate DAPI-stained nucleus and FITC reflect the bound mAbs. **B.** ELISA curves of HRP-labeled mAbs binding to either fiber 1 protein or FAdV-4. Each point stands for mean values of 3 replicates and error bar: ± SEM.

To validate the binding capacity of the expressed mAbs to FAdV-4, IFA was conducted. As shown in Fig. 2A, all five mAbs could specifically recognize and bind to FAdV-4 *in vitro*. Moreover, the HRP-labeled mAbs demonstrated perfect binding curves with both fiber 1 protein and FAdV-4 (Fig. 2B). These results collectively demonstrate that the screened 5 mAbs could specifically bind to both FAdV-4 and fiber 1 protein.

### Antibody pairing for sandwich ELISA

**Fig. 3:**
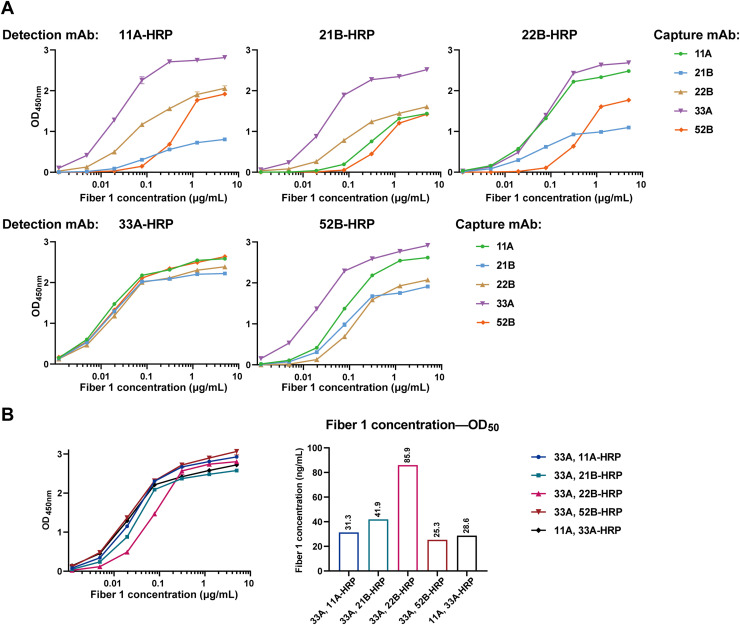
Determination of optimal mAb pair. A. ELISA curves of the antibody pairing using the 5 mAbs. Each graph shows the results of the fixed detection antibody and different capture mAbs. **B.** ELISA curves of the five best mAb pairs on one plate (Left) and the fiber 1 concentration reaching OD_50_ of each pair (Right). The error bar: ± SEM (*n* = 3). The fiber 1 concentration (ng/mL) reaching OD_50_ is noted above each bar.

To determine the best pair of mAbs for fiber 1 protein-specific sandwich ELISA, the 5 mAbs were tested in pairs. All curves showed a classical logarithmic pattern, indicating the specific binding occurred (Fig. 3). According to the curves in Fig. 3A, the best mAb pairs were 33A and 11A-HRP, 33A and 21B-HRP, 33A and 22B-HRP, 11A and 33A-HRP, 33A and 52B-HRP in each plate. To determine the best pair, the 5 pairs were tested in the same plate. The result illustrated that OD_max_ values and sample concentrations reaching OD_50_ were similar, while the pair of 33A and 52B-HRP showed a slight advantage with the detection limit of 25.3 ng/mL fiber 1 protein (Fig. 3B).

**Fig. 4:**
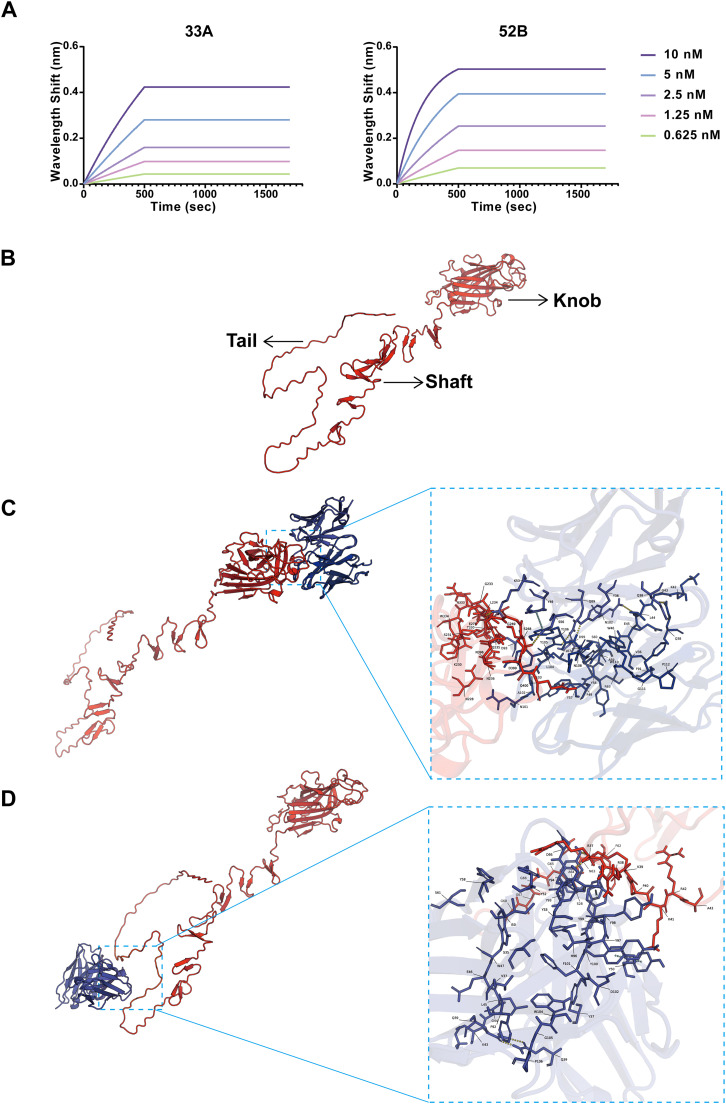
Characterization of the binding between mAbs and fiber 1 protein. A. Binding kinetics of 33A and 52B to fiber 1 protein by BLI. **B.** Illustrative image of the fiber 1 protein 3D structure generated by AlphaFold2-multimer and PyMOL. **C.** The docking model of 33A binding to fiber 1 protein. Fiber 1: red, and 33A: blue. **D.** The docking model of 52B binding to fiber 1 protein. Fiber 1: red, and 52B: blue. The bonds involved in the interaction between mAbs and fiber 1 were marked in different colors: hydrogen bonds in yellow, ionic bonds in purple and pi-conjugated bonds in green.

Next, the binding affinities of 33A and 52B to fiber 1 protein were assessed by BLI, which were both less than 10^-^^12^ M (Fig. 4A and Table 2). The extremely high affinities between mAbs and fiber 1 contributed to the low detection limit of the mAb pair. Moreover, the interactions of mAbs with fiber 1 protein were simulated by AlphaFold2-multimer. The structure of fiber 1 protein was firstly illustrated, showing clear knob, shaft and tail regions as described previously (Song et al., 2024) (Fig. 4B). Then, the fiber 1-mAbs binding surfaces were predicted by docking the 33A and 52B to fiber 1 protein, respectively. As shown in Fig. 4C and 4D, 33A specifically recognized the residues in the C terminus (knob domain), while 52B bound to the N terminus (tail domain). The two epitopes were spatially distinct without sequential nor steric overlap, proving this pair of mAb was suitable for fiber 1 detection. Combining with the high affinities, 33A and 52B formed the ideal pair for fiber 1-specific sandwich ELISA.

**Table 2:** The affinities of 33A and 52B to fiber 1 protein.

mAb	K_on_ (1/Ms)	K_off_ (1/s)	K_D_ (M)
33A	1.011 × 10^5^	<1 × 10^−7^	<1 × 10^−12^
52B	4.722 × 10^5^	<1 × 10^−7^	<1 × 10^−12^

### Optimization of the sandwich ELISA to detect fiber 1 protein

**Fig. 5:**
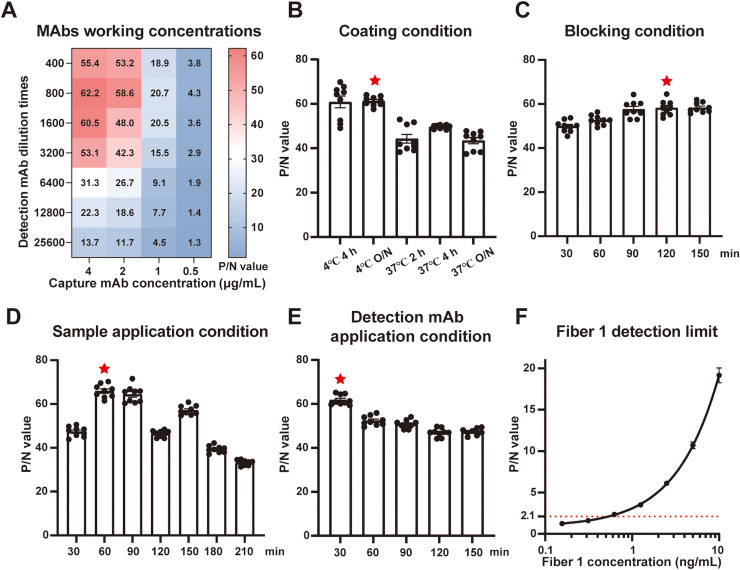
Optimization of the sandwich ELISA to detect fiber 1 protein. A. Heat map to determine the optimal capture and detection mAbs concentrations. **B.** Determination of coating condition. O/N represents overnight. **C.** Determination of optimal blocking condition. **D.** Determination of optimal sample application condition. **E.** Determination of optimal detecting mAbs incubating period. The condition that reaches maximum P/N value is defined as optimal, which is noted by a red asterisk. The error bar: ± SEM (*n* = 9). **F.** The sensitivity of the established ELISA to detect fiber 1 protein. The detection limit is defined as the fiber 1 concentration at a P/N value of 2.1 (red line). The error bar: ± SEM (*n* = 3).

Firstly, the concentrations of capture (33A) and detection (52B-HRP) mAbs were optimized. When the capture mAb was 4 μg/mL and the detection mAb was diluted 1:800, the P/N value was highest (Fig. 5A). Next, the coating condition was optimized. The highest P/N value was obtained when capture mAb was coated at 4°C overnight as shown in Fig. 5B. Regarding the blocking condition, the P/N values of 120 min and 150 min were comparable, therefore 120 min of blocking was determined as optimum (Fig. 5C). Moreover, the highest P/N value was achieved when sample (fiber 1 protein) was incubated for 60 min at room temperature (Fig. 5D). Lastly, P/N value was the highest when detection mAb was incubated for 30 min (Fig. 5E). Under these conditions, the sandwich ELISA could detect 0.532 ng/mL fiber 1 protein as shown in Fig. 4F. Taken together, the optimized conditions for fiber 1-specific ELISA were determined and the detection limit was less than 1 ng/mL.

### The established ELISA showed high specificity to FAdV-4

**Fig. 6:**
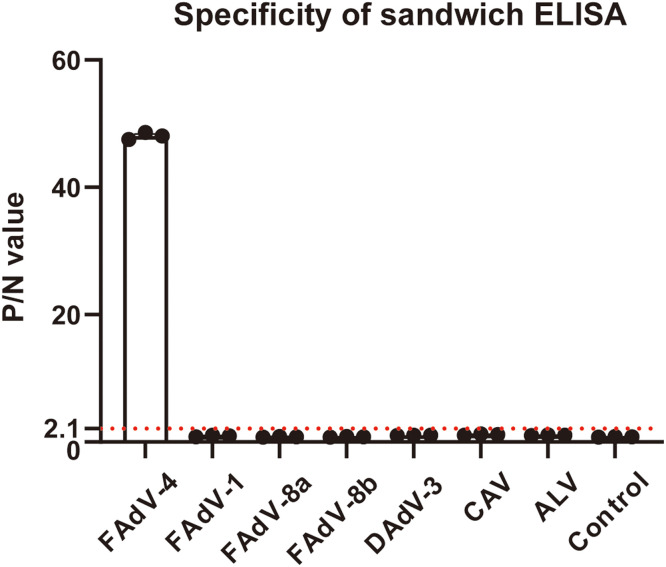
Specificity of the sandwich ELISA. Viral sample that results in P/N > 2.1 is regarded as positive. The error bar: ± SEM (*n* = 3).

To assess the specificity of the established ELISA, several common avian viruses were examined in the assay. FAdV-4, instead of FAdV-1, FAdV-8a, FAdV-8b, DAdV-3, CAV, nor ALV, was detected as shown in Fig. 6, suggesting the assay exhibited excellent specificity.

### The established ELISA exhibited good repeatability and reproducibility

Then, the repeatability and reproducibility of the assay were tested, which were reflected by the intra-batch and inter-batch CV, respectively. The results showed that both intra-batch (2.65 %∼4.73 %, Table 3) and inter-batch (2.61 %∼4.91 %, Table 4) CV were lower than 5 %, meeting the criteria of great repeatability and reproducibility.

**Table 3:** Repeatability of the sandwich ELISA.

Repetitions	Sample				
	1	2	3	4	5
1	1.988	0.931	1.070	1.998	1.408
2	1.967	0.976	1.142	2.111	1.495
3	2.009	0.956	1.143	2.068	1.430
4	2.024	0.973	1.124	1.995	1.484
5	2.074	1.006	1.175	2.040	1.517
6	2.100	1.008	1.261	2.045	1.561
7	2.013	1.021	1.159	1.960	1.467
8	2.122	1.042	1.186	1.958	1.439
X	2.037	0.989	1.157	2.022	1.475
SD	0.055	0.037	0.055	0.054	0.050
CV (%)	2.70 %	3.71 %	4.73 %	2.65 %	3.39 %

**Table 4:** Reproducibility of the sandwich ELISA.

Repetitions	Sample				
	1	2	3	4	5
1	1.948	1.013	1.086	1.998	1.517
2	2.065	0.975	1.143	2.157	1.519
3	2.028	0.940	1.060	1.973	1.451
X	2.014	0.976	1.096	2.043	1.496
SD	0.060	0.037	0.042	0.100	0.039
CV (%)	2.96 %	3.75 %	3.83 %	4.91 %	2.61 %

### The established ELISA could diagnose FAdV-4 in clinical serum samples

**Fig. 7:**
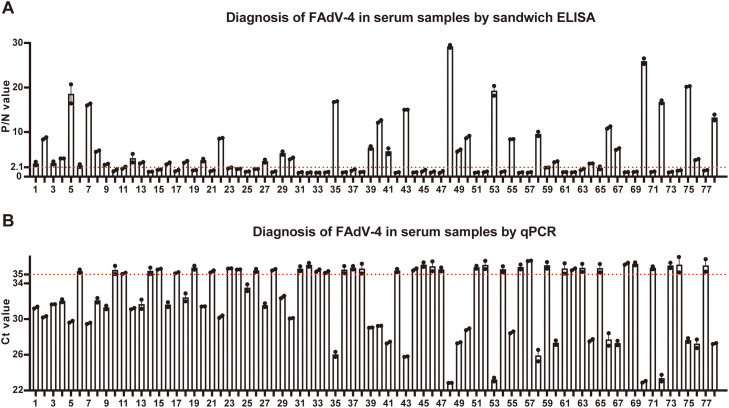
Diagnosis of FAdV-4 in clinical serum samples by the established sandwich ELISA and qPCR. A. P/N values of 78 clinical serum samples by sandwich ELISA. The red line: P/N = 2.1, which is the threshold of FAdV-4 positivity. The error bar: ± SEM (*n* = 2). **B.** Ct values of qPCR results detecting FAdV-4 in 78 clinical serum samples. The red line: Ct = 35, which is the threshold of FAdV-4 positivity. The error bar: ± SEM (*n* = 2).

To examine whether the ELISA could diagnose clinical samples, 78 serum samples were tested. There were 37 sera turned to be positive and 39 samples be negative by both ELISA and qPCR (Fig. 7). On the other hand, one serum showed positive readout in ELISA yet negative in qPCR and another showed negative readout in ELISA yet positive in qPCR. Anyway, the ELISA results showed 97.44 % agreement with the qPCR results and the kappa value was 0.949 (Table 5).

**Table 5:** Overview of FAdV-4 diagnosis in clinical serum samples by sandwich ELISA and qPCR.

		Sandwich ELISA			Coincidence rate	Kappa
		Positive	Negative	Total		
**qPCR**	Positive	37	1	38	97.44 %	0.949
	Negative	1	39	40		
	Total	38	40	78		

### The established ELISA could quantify the viral antigens *in vivo*

**Fig. 8:**
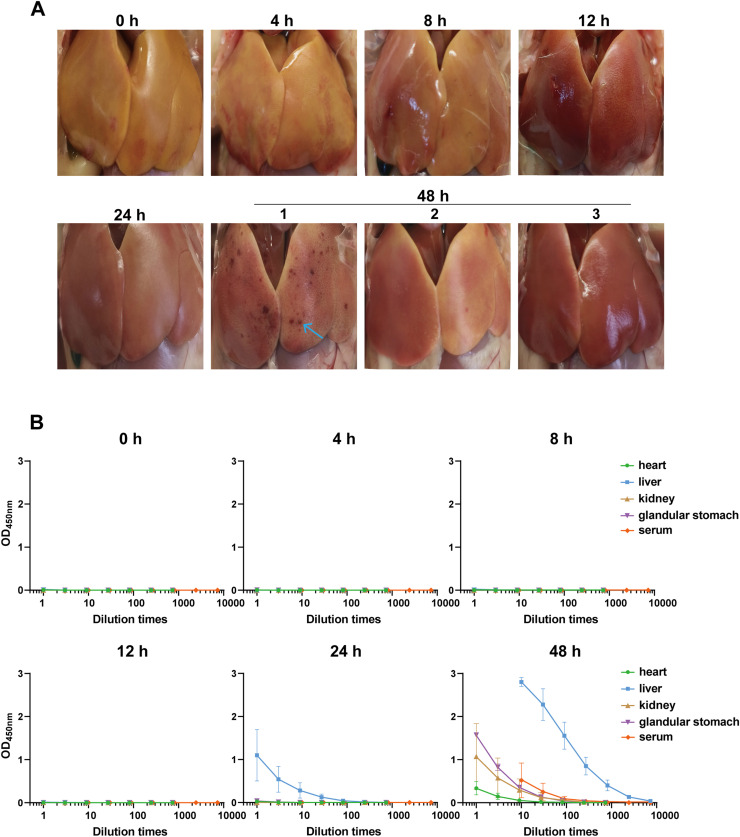
FAdV-4 kinetics in chick tissues after infection. A. The liver images at different time points after FAdV-4 infection. Blue arrow points at necrotic foci in the liver of a chicken that died at 47 hpi. **B.** Detection of viral antigen in the heart, liver, kidney, glandular stomach and serum from infected chicks by ELISA. Each point meant the mean values of 3 chicks, and the error bar: ± SEM.

To test whether the assay could detect FAdV-4 in tissues, 6-day-old chicks were infected with the virus. Livers, hearts, kidneys, glandular stomachs and sera were collected from 3 chicks at 4, 8, 12, 24 and 48 hours post infection (hpi) respectively. All animals were sacrificed at the right time point except one was dead at 47 hpi, which was grouped in 48 hpi. Its liver showed necrotic spot, which was not found in other animals (Fig. 8A). All tissue homogeneous supernatants and sera were applied to the ELISA to detect the FAdV-4, which were below the detection limit until 24 hpi except for livers. In contrast, FAdV-4 was detected in kidneys, stomachs, hearts and sera at 48 hpi, although clearly lower than those in livers (Fig. 8B).

**Fig. 9:**
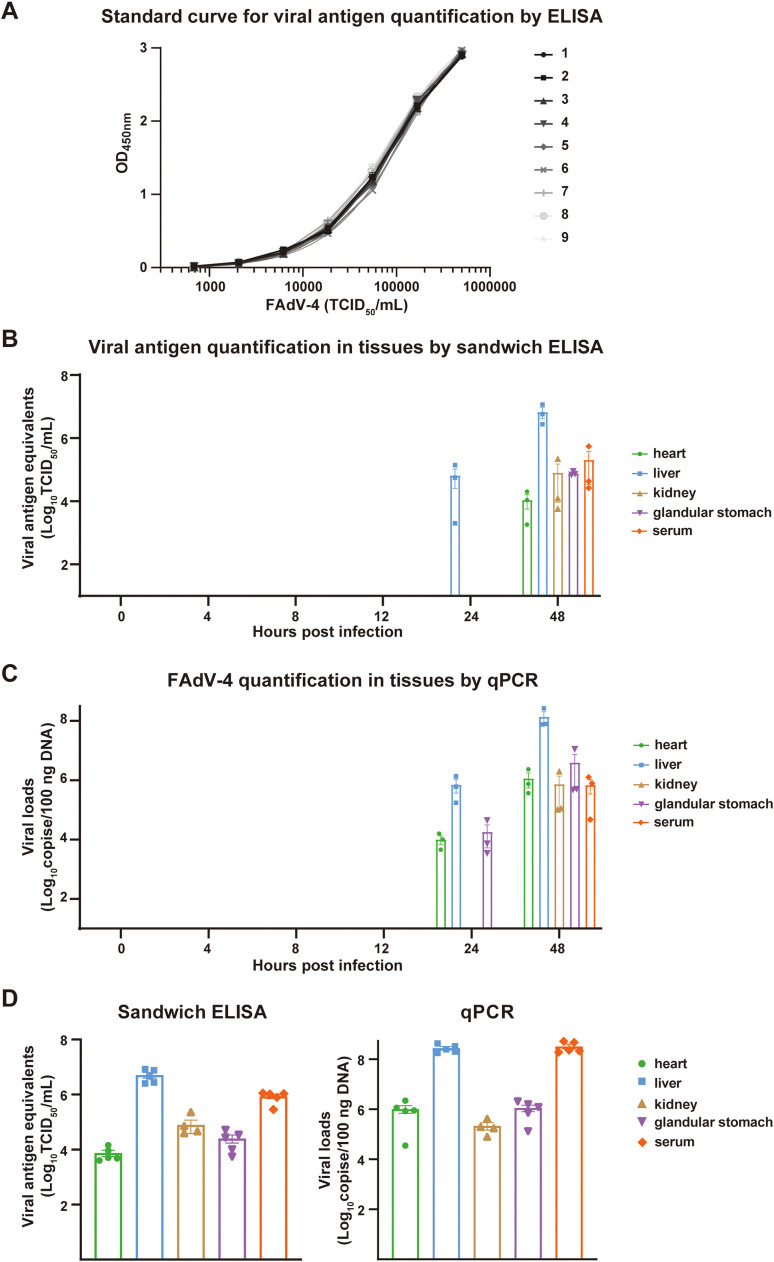
Comparison of FAdV-4 antigen quantification in chick tissues between ELISA and qPCR. A. Standard curve of the sandwich ELISA for viral antigen quantification. The results of 9 individual replicates were plotted. **B.** Viral antigen quantification results in the infected chick tissues evaluated by sandwich ELISA. **C**. Viral quantification in the infected chick tissues evaluated by qPCR. **D.** Viral antigen quantification results in dead chick tissues evaluated by sandwich ELISA and qPCR. Each point represented 1 chick, and the error bar: ± SEM.

Next, we would like to quantify the viral antigen levels in tissues using the established sandwich ELISA. Therefore, a standard ELISA curve using FAdV-4 was drawn as shown in Fig. 9A, which exhibited a perfect S-shaped curve with an excellent repeatability. The limit of the assay to detect FAdV-4 was 1520 TCID_50_/mL. Consistent with the expectation, the viral antigen was below the detection limit in all tissues at 4, 8, and 12 hpi. At 24 hpi, FAdV-4 was solely detected in livers by ELISA (Fig. 9B). More antigens were detected in livers at 48 hpi, which was significantly higher than those in other tissues. To validate the findings in ELISA, qPCR was performed in parallel. Apart from the detectable virus in hearts and stomachs at 24 hpi, all qPCR results showed the same trends as ELISA (Fig. 9C). Furthermore, viral antigen levels in tissues of dead chicks were examined as well. Similarly, livers bore the highest viruses, which was consistent with the results from qPCR (Fig. 9D). These results collectively indicated the ELISA could be an alternative for FAdV-4 quantification.

## Discussion

Effective viral prevention and treatment strategies highly depend on a precise diagnosis. Although qPCR is the gold standard for FAdV-4 diagnosis, methods that targeting viral proteins are needed as well. Sandwich ELISA based on mAbs are widely used for protein detection, whereas the FAdV-4 diagnostic ELISA kit is not available. Studies showed that mAbs specific for fiber 1 or fiber 2 were able to detect FAdV-4 with a detection limit of 12.5 ng/mL fiber proteins (Shao et al., 2019b). In our study, the detection limit of the ELISA was lower than 1 ng/mL, demonstrating excellent sensitivities.

The mAbs used in this work were derived from rabbits, which possess remarkably large antibody repertoires and high affinities (Ros et al., 2020). Normally, affinities of mature mouse antibodies are 10^−9^∼10^−10^ M, while the affinities of 33A and 52B antibodies are less than 10^−12^ M, validating the idea that rabbit antibodies have higher affinities than those from mice (Nguyen et al., 2018; Li et al., 2023). With the high affinities of these two mAbs to fiber 1 protein, the detection limit of the ELISA was lower than 1 ng/mL. There’s no ELISA kit available in the market for FAdV-4 diagnosis, but the ELISA kit for ALV diagnosis is able to detect 0.2 ng/mL p27 protein (Wei et al., 2024). Furthermore, the epitopes recognized by 33A and 52B were located at the opposite terminus of fiber 1 protein, separated by a long shaft domain. This spatial distance facilitated the binding of capture and detection mAbs to fiber 1 protein. Thus, the established assay is potential to be a useful tool for FAdV-4 diagnosis.

There’s no doubt that the qPCR is the standard for FAdV-4 diagnosis. However, the DNA extraction requirement complicates and prolongs the viral detection procedure. In contrast, tissue or serum samples could be directly applied to ELISA, which is an obvious advantage over qPCR. On the other hand, viral protein detection is more straightforward than viral gene detection despite both being specific. The serum samples in this study were tested by both qPCR and ELISA, which showed 97.44 % consistency. The negligible disagreement was probably due to the sample processing difference. Taken together, ELISA stands for a great diagnosis tool for FAdV-4.

In addition, the established ELISA not only could identify the FAdV-4, but also could quantify the viral antigens *in vivo*. Although the overall patterns of viral quantification in tissues by ELISA and qPCR were similar, the exact viral antigen amounts were distinct. We speculate the reason might be the different calculation methods. To our knowledge, the minimum viral counts to cause pathogenicity in chicks varies depending on FAdV-4 virulence, host immune status and environmental conditions (Wang and Zhao, 2019). Consequently, the key challenge of commercializing the FAdV-4 ELISA is the standards of a clear cutoff to distinguish positive and negative samples.

Empirically, chicks younger than 7-day-old suddenly die from FAdV-4 infection between 48 hpi to 72 hpi (Schachner et al., 2018). Consistently, the infected animals in our study died between 47 hpi to 72 hpi, indicating our challenge model was reliable. On the other hand, the survived animals showed no clinical signs, suggesting the overload of virus might be the cause of host mortality. It has been reported that liver is the primary target for FAdV-4, whereas the virus was detected in tissue homogenates of other organs including heart, kidney, spleen and lung as well (Mo et al., 2019; Sun et al., 2019). Our results showed that FAdV-4 antigen in livers were the highest, which is consistent with the previous conclusion that livers were the primary targets for FAdV-4 (Li et al., 2018; Yuan et al., 2021).

In summary, a sandwich ELISA based on rabbit mAbs for FAdV-4 diagnosis was established and optimized. We systemically examined the optimized assay, which exhibited outstanding specificity, repeatability, reproducibility and low detection limit as 0.532 ng/mL fiber 1 protein. Importantly, the viral antigen in clinical sera and tissues could be quantified by this assay, facilitating the epidemic research and viral prevention.

## Ethics statement

All animal experiments were approved by the Animal Care and Use Committee, Anhui Agricultural University (AHAU 2022043, for rabbits and AHAU 2024047, for chickens). All animal handling and experiments were performed strictly in accordance to the guidance and regulations of the Animal Care and Use Committee, Anhui Agricultural University.

## Funding

This work was funded by the National Key Research and Development Plan (2022YDF1801800, to Lisha Zha), Anhui Provincial Natural Science Foundation (2408085QC074, to Xinyue Chang) and Starting Foundation of Anhui Agricultural University (to Xinyue Chang).

## CRediT authorship contribution statement

**Huimin Ma:** Visualization, Methodology, Formal analysis, Data curation. **Yuhang Zhou:** Resources, Methodology. **Shipeng Wang:** Visualization, Software. **Xiangyu Xie:** Resources. **Ruiji Chen:** Resources. **Qi Zheng:** Writing – review & editing. **Lisha Zha:** Writing – review & editing, Supervision, Investigation, Funding acquisition. **Xinyue Chang:** Writing – original draft, Funding acquisition, Conceptualization.

## Disclosures

Lisha Zha is involved in a pet vaccine company and owns shares. Other authors claim no interest conflict.

## Data Availability

All data generated or analyzed during this study are included in this published article.
